# Supplementary material for: Platelet Reactivity and Cardiovascular Mortality Risk in the LURIC Study
Source: J Clin Med. 2023 Feb 28;12(5):1913. doi: 10.3390/jcm12051913 (PMC10003439; doi:10.3390/jcm12051913)
Supplement: Supplementary file 1 [file jcm-12-01913-s001.zip › jcm-2047139-supplementary.pdf]

Supplementary Material

1. Extended description of Patient characteristics according to the LURIC study[1]:

Coronary artery disease (CAD) was diagnosed based on clinical signs and coronary angiography. Clinically relevant CAD was defined as the occurrence of ≥1 stenosis of ≥20% in ≥1 of 15 coronary segments. Individuals with stenoses < 20% were considered not to have CAD.

Stroke was defined clinically by documented history of a previous cerebrovascular disease event (transient ischaemic attack, prolonged ischaemic neurological deficit, cerebral infarction with or without a remaining neurological deficit) or by documented carotid plaques (≥50% luminal obstruction).

Peripheral vascular disease was defined by a history of intermittent claudication, angiographic documentation of atherosclerotic luminal obstruction of the peripheral arteries or a history of a peripheral arterial intervention for atherosclerotic disease (angioplasty or surgery).

Acute coronary syndrome (ACS) was defined as the spectrum of clinical conditions ranging from UA to non-ST-elevation (primarily non-Q-wave) and ST-elevation (primarily Q-wave) MI.

- Unstable angina was defined by subjective assessment of clinical symptoms according to the severity of the clinical manifestation using Braunwald's classification[2]
- It is of note that LURIC was performed before the definition of non-ST-elevation Myocardial infarction. Therefore MI was stratified by non Q wave and Q wave infarction.
    - Definite Q-wave MI was diagnosed if at least one of the two non-ECG criteria were fulfilled: either typical symptoms, or typical enzyme elevation and a pathologic Q-wave had evolved in the resting ECG. Typically, a definite Q-wave MI presents initially as ST-elevation MI, but a definite Q-wave MI may also evolve after a diagnosis of definite non-ST-elevation MI
    - Definite non-Q-wave MI was defined as an MI that met both of the non-ECG criteria for MI reported above (typical symptoms and typical enzyme changes). Typically, a definite non-Q-wave MI evolves after non-ST-elevation MI. However, a non-Q-wave MI may also evolve in some individuals after ST-elevation MI.

Arterial hypertension was defined as systolic bloodpressure > 140mmHg and/or diastolic Bloodpressure < 90 mmHg.

History of PTCA was defined as any coronary intervention proven by medical records before study inclusion.

Dyslipidemia was defined as total cholesterol ≥ 240 mg/dl or triglycerides ≥ 150 mg/dl or HDL cholesterol ≤ 35 mg/dl males, ≤ 45 mg/dl females; isolated low HDL cholesterol (= HDL cholesterol ≤ 35 mg/dl males, ≤ 45 mg/dl females in the presence of LDL-cholesterol < 130 mg/dl and triglycerides < 200 mg/dl) was not counted (frequency for all subjects regardless of treatment with or without lipid-lowering agents/prevalence in untreated subjects).

Alcohol intake was self reported and defined as any regular alcohol intake irrespective of wine, beer and liquor.

Smoking was self reported and defined as any smoking irrespective of Pack-years.

Chronic kidney disease was defined as an eGFR < 60ml/min according to the CKD-Epi Formula[3]

Cancer was specified based on patient history. If a patient was diagnosed with a malignant disease within the previous 5 years, this resulted in study exclusion.

Diabetes mellitus was defined as fasting (no caloric intake within last 8 h) plasma glucose ≥ 126 mg/dl (≥ 7.0 mmol/l) or 2 h post glucose load ≥ 200 mg/dl (≥ 11.1 mmol/l) or both, or symptoms of diabetes (polyuria, polydipsia and unexplained weight loss) plus casual plasma glucose ≥ 200 mg/dl (casual = sample taken any time of the day). In addition, HbA1c ≥ 6.5% was considered as diagnostic for diabetes.

Medication usage was self reported and recorded as disclosed by the patients. The medication history included the brand name, dose, duration and time of last intake (in reference to the time of blood sampling for LURIC) of any medication taken within the previous four weeks (Appendix IV). Great efforts were made to record such information as accurately as possible. This included verification of data in patients charts, comparison with prescription schedules or repeat telephone calls to the patient in case of missing or discrepant data.

Anti-platelet therapy
To prevent false-positive classification of ADP-induced platelet reactivity, patients on the $P2Y_{12}$ inhibitor clopidogrel (N=104, 5.8%) were excluded from the study. In total, 1046 (68.82%) patients were on 75 – 100mg aspirin. There was no statistical difference between the groups ($p = 0.9799$).

1.1. Laboratory analysis

All blood samples were drawn on the day coronary angiography was scheduled. Blood count was measured on an automated analyser Technicon H-1 (Technicon, Bad Vilbel Germany) and Advia 120 (Bayer Diagnostics, Tarrytown, USA). Glucose was measured enzymatically on a Hitachi 717 analyzer (Roche, Mannheim, Germany). Glycated hemoglobin was measured with an immunoassay (Hemoglobin A1c UNIMATE 5; Hoffmann-LaRoche, Grenzach-Whylen, Germany). Lipoproteins were separated using a combined ultracentrifugation–precipitation method and measured on a WAKO 30 R analyzer (WAKO Chemicals GmbH, Neuss, Germany). Triglycerides were quantified with an enzymatic assay on a Hitachi 717 analyzer (Roche Diagnostics, Mannheim, Germany). Creatinine was measured with the Jaffé method

on a Hitachi 717 analyzer (Roche). eGFR was calculated according to the chronic kidney diseases epidemiology collaboration equation. High-sensitive CRP (hsCRP) was measured with an nephelometric assay (N LATEX CRP mini kit Behring nephelometer II, Dade Behring GmbH, Marburg, Germany).

Reference:

1. Winkelmann BR, Marz W, Boehm BO, et al. 2001.Rationale and design of the LURIC study--a resource for functional genomics, pharmacogenomics and long-term prognosis of cardiovascular disease. *Pharmacogenomics*.2(1 Suppl 1):S1-73.
2. Braunwald E. 1989.Unstable angina. A classification. *Circulation*.80(2):410-414.
3. Levey AS, Stevens LA, Schmid CH, et al. 2009.A new equation to estimate glomerular filtration rate. *Ann Intern Med*.150(9):604-612.

2. Statistical Methods

In any case where Cox regression has been performed, the *proportional hazards assumption* is tested in terms of *scaled Schönfeld residuals* by the R function *cox.zph*.

The details of the calculation of the cut-off values cut-off1 < cut-off2 specifiying *Low Platelet reactivity group*, *High Platelet reactivity group* and the *Reference group* - from now on referred to as *CD63 categories* - for Platelet reactivity based on Cox model based hazard ratio estimations by restricted cubic splines (RCS) are given in subsection *Splines for HR estimation* below.

2.1 Baseline characteristics and medication

For metric covariates, the p-values refer to differences between CD63 categories and are derived from the Kruskal-Wallis rank sum tests (with the aid of the R function kruskal.test) for testing that the location parameters of the distribution of the considered metric variable are the same in each CD63 category. For binary variables, the p-values are calculated with the aid of the R function fisher.test (two-sided Fisher test) for testing the null hypothesis of independence. For binary (resp. metric) variables, the post-hoc analyses were performed with the aid of pairwise Fisher tests resp. Wilcoxon rank sum tests by the R functions pairwise.fisher.test (library fmsb) resp. pairwise.wilcox.test (library stats); therein, the p value is adjusted by the Holm method.

2.2 Estimation of hazard ratios (HRs) for *cardiovascular death* and *all-cause death* in terms of CD63 categories

The estimated HRs are calculated with the aid of the R function coxph, the 95% CI for the HRs are given by exp(coef ± 1.96 x standard model error of coef), where coef denotes the coefficient for the respective covariate in the risk function. In order to control multicollinearity effects, we compute for any solution of these Cox regression models the variance inflation factors (VIFs) with the R function vif. In estimating HRs for cardiovascular death and all-cause death in terms of CD63 categories (adjusted

by Model 0 – Model 4) the VIFs are all in the interval [1, 1.6) and indicate that the Cox models are well-conditioned.

2.3 Comparison of the risk factor CAD at baseline time with altered Platelet reactivity

p-values regarding this comparison refer to the log-rank test (calculated with the aid of the R functions survdiff and pchisq).

2.4 Explained relative risk by CD63 category

For any CD63 category, the explained relative risk is calculated with the aid of the R function coxphERR of the library clinfun by applying the R function coxphERR resampling 2000 times without replacement with N_samp=round(2N_tot/3), where N_tot is the total number of patients in the considered CD63 category. The percentiles in this regard refer to these 2000 random selections.

2.5 Restricted cubic splines (RCS) for HR estimation

RCS are set up to have five knots at the 5th, 27.5th, 50th, 72.5th and 95th percentile of CD63 (resp. CD62). Based on these percentiles, the base splines for RCS (defined by these knots) are calculated with the aid of the R function rcs.

We performed 2000 random selections without replacement of round(2N_tot/3) from N_tot elements with the aid of the R function sample.int, where N_tot is the total number of patients in the considered population and calculate for any of these 2000 random selections the Cox regression coefficients of the aforementioned fixed base splines with the aid of the R function coxph.

The solid lines (lower (resp. upper) boundary of the blue region) are calculated via resampling and correspond to the median (resp. 2.5th (resp. 97.5th) percentile) RCS values referring to the aforementioned 2000 random selections.

Based the 2.5th percentile RCS values (represented by the lower boundary of the blue region), for CD63, endpoint cardiovascular death, Model 2, the value cut-off1 (resp. cut-off2) is defined to be the highest (resp. lowest) value such that the mean logarithmic of the values of the 2.5th percentile curve over the interval (1,cut-off1] (resp. [cut-off2,max(CD63)]) is larger than zero.

3. Figure S1: Identification of High and Low platelet reactivity

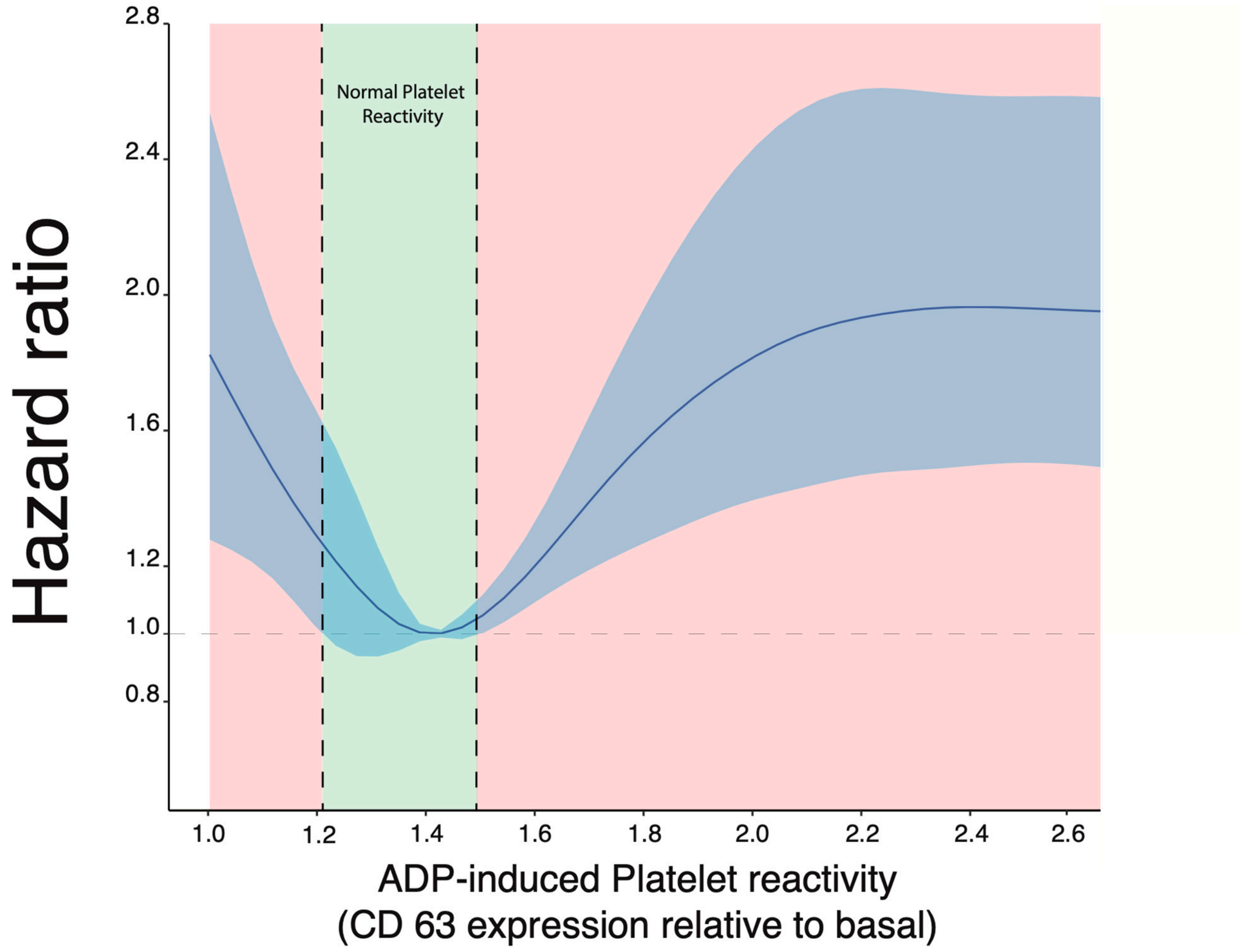

Figure S1: ADP-induced CD63-expression was modelled with the aid of restricted cubic splines for the hazard of cardiovascular mortality. The blue area denotes the 95% confidence interval. Threshold was chosen when the confidence interval significantly deviated from 1.0.

4. **Figure S2: Basal-CD63 expression of platelets according to ADP-responsiveness**

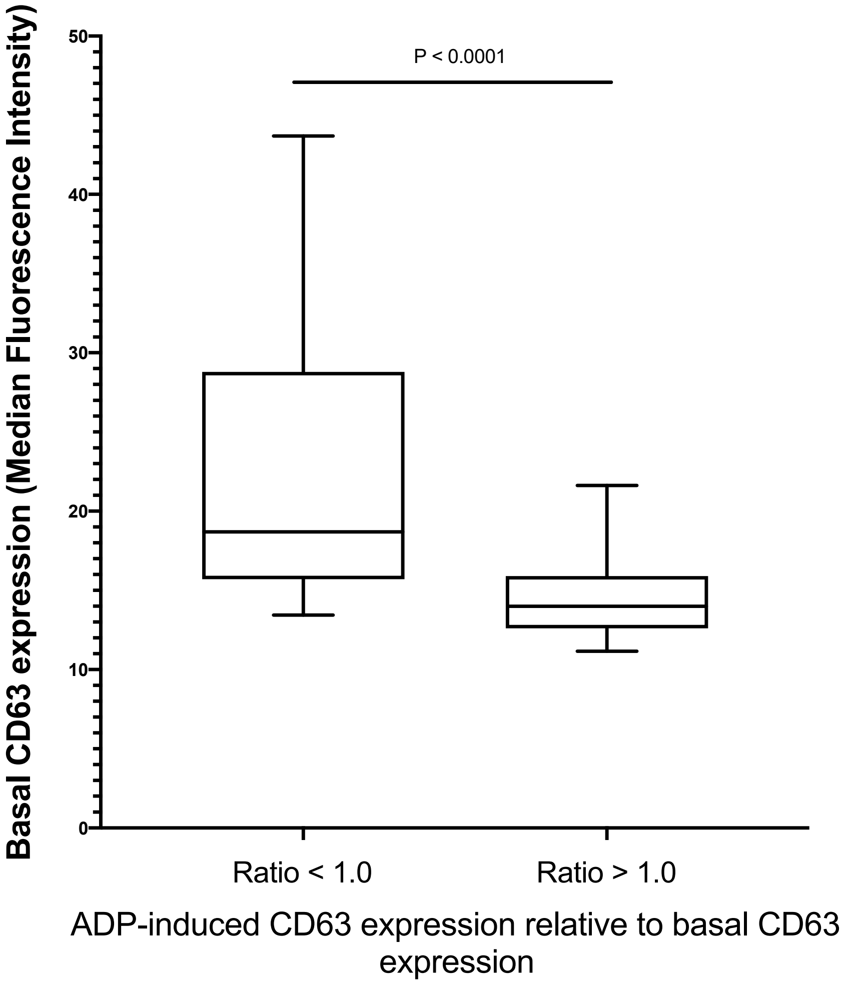

Figure S2: Boxplot of platelet CD63-expression stratified for ADP-response. Platelets that were unresponsive to ADP-stimulation (defined as: ADP-induced CD63 expression/ Basal CD63 expression < 1.0) had significantly higher basal CD63 expression as an indicator of preactivation. Therefore, these samples (N=156, 8.7%) were excluded from the analysis.

5. **Figure S3: ADP-induced platelet reactivity and cardiovascular and all-cause mortality in the LURIC study – CD62P, CD63 expression and Fibrinogen-binding**

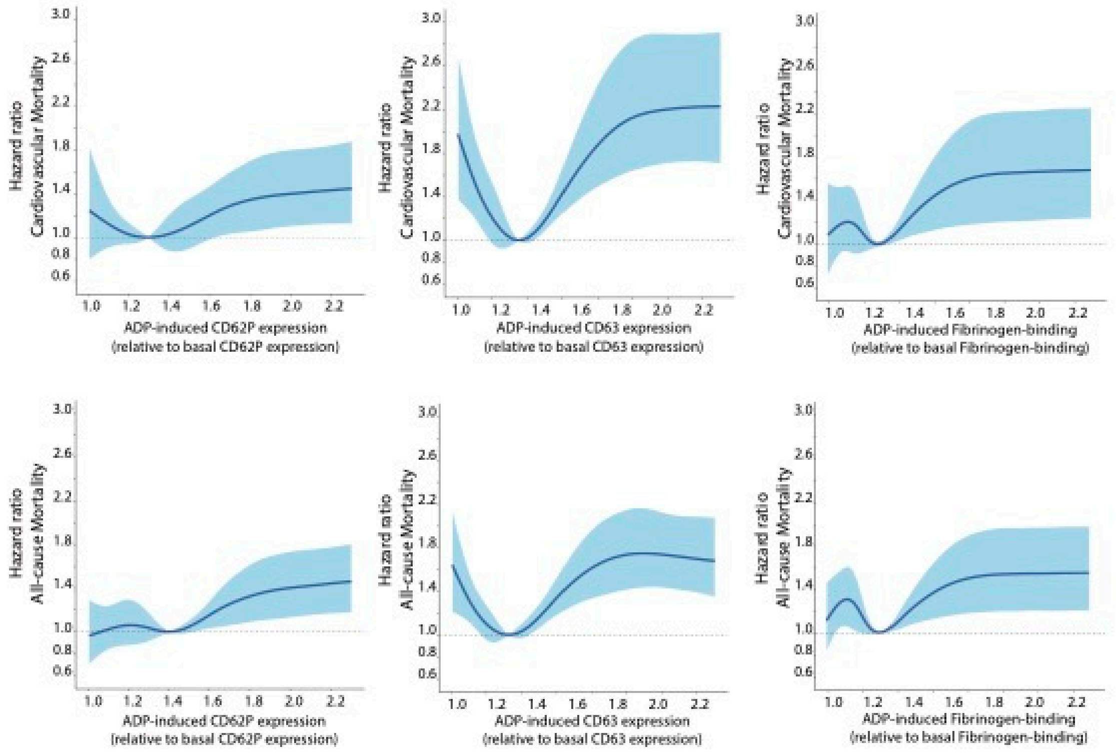

Figure S3: ADP-induced CD62P and CD63-expression and Fibrinogen-binding was modelled with the aid of restricted cubic splines for the hazard of cardiovascular- and all-cause mortality. The blue area denotes the 95% confidence interval.

6. **Figure S4: TRAP-induced platelet reactivity and cardiovascular and all-cause mortality in the LURIC study – CD62P, CD63 expression and Fibrinogen-binding**

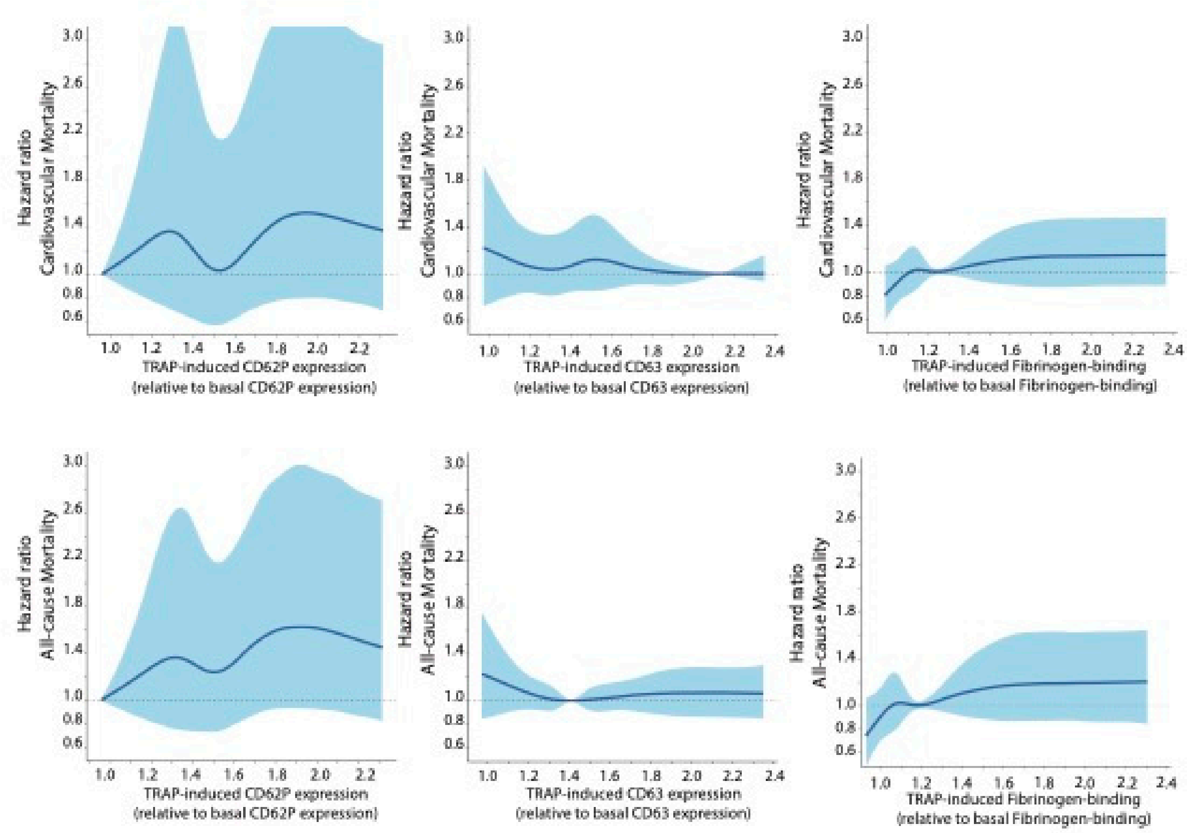

Figure S4: TRAP-induced CD62P and CD63-expression and Fibrinogen-binding was modelled with the aid of restricted cubic splines for the hazard of cardiovascular- and all-cause mortality. The blue area denotes the 95% confidence interval.

Figure S5: Survival curves for High and Low Platelet reactivity with respect to coronary artery disease – cardiovascular mortality

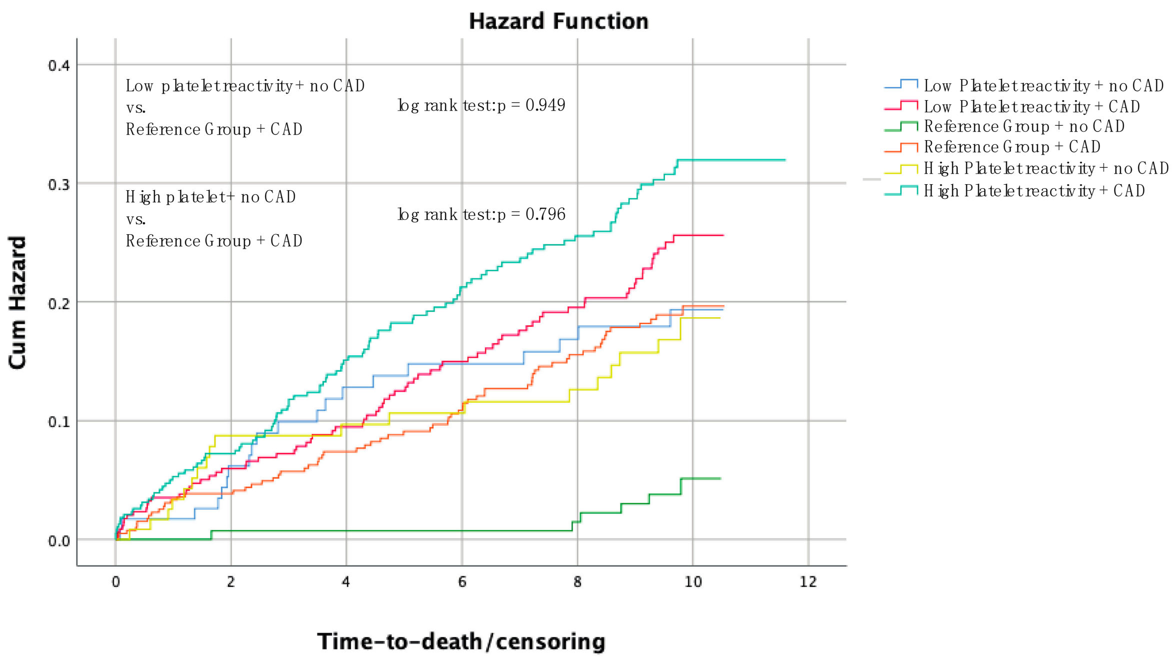

Figure S5: Kaplan-Meier cumulative event curves for cardiovascular mortality in patients stratified by presence and absence of coronary artery disease and High and Low Platelet reactivity respectively.

Figure S6: Survival curves for High and Low Platelet reactivity with respect to coronary artery disease – cardiovascular mortality

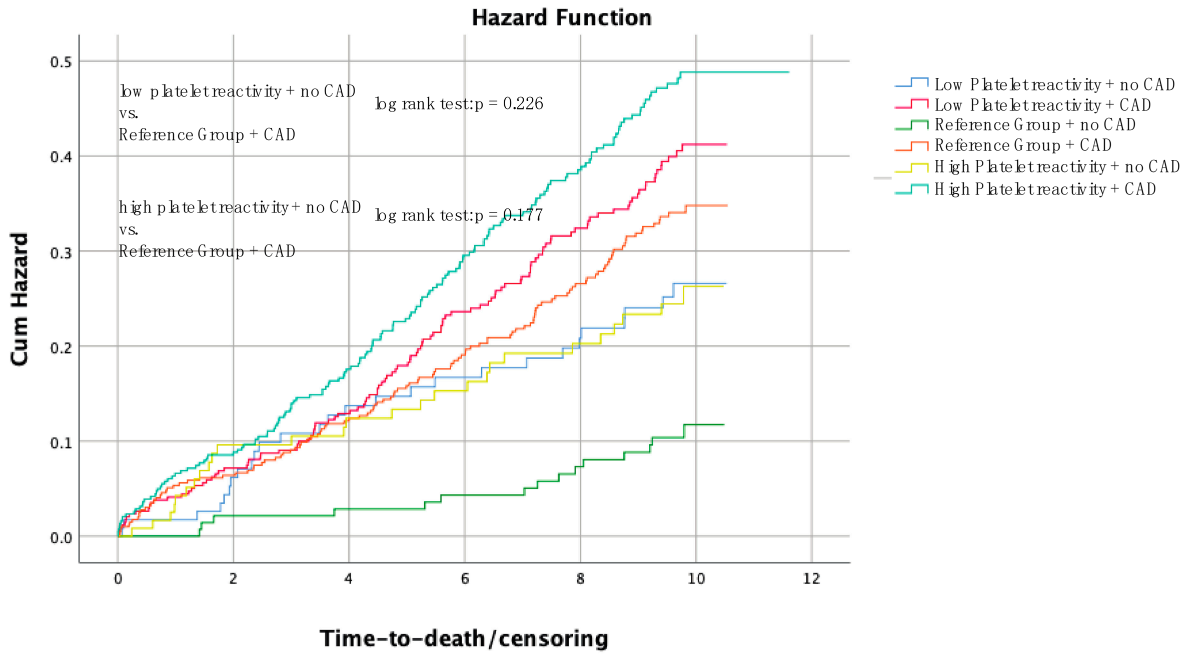

Figure S5: Kaplan-Meier cumulative event curves for mortality in patients stratified by presence and absence of coronary artery disease and High and Low Platelet reactivity respectively.

Figure S7: CD62P and CD63 expression of platelets in response to ADP and TRAP stimulation

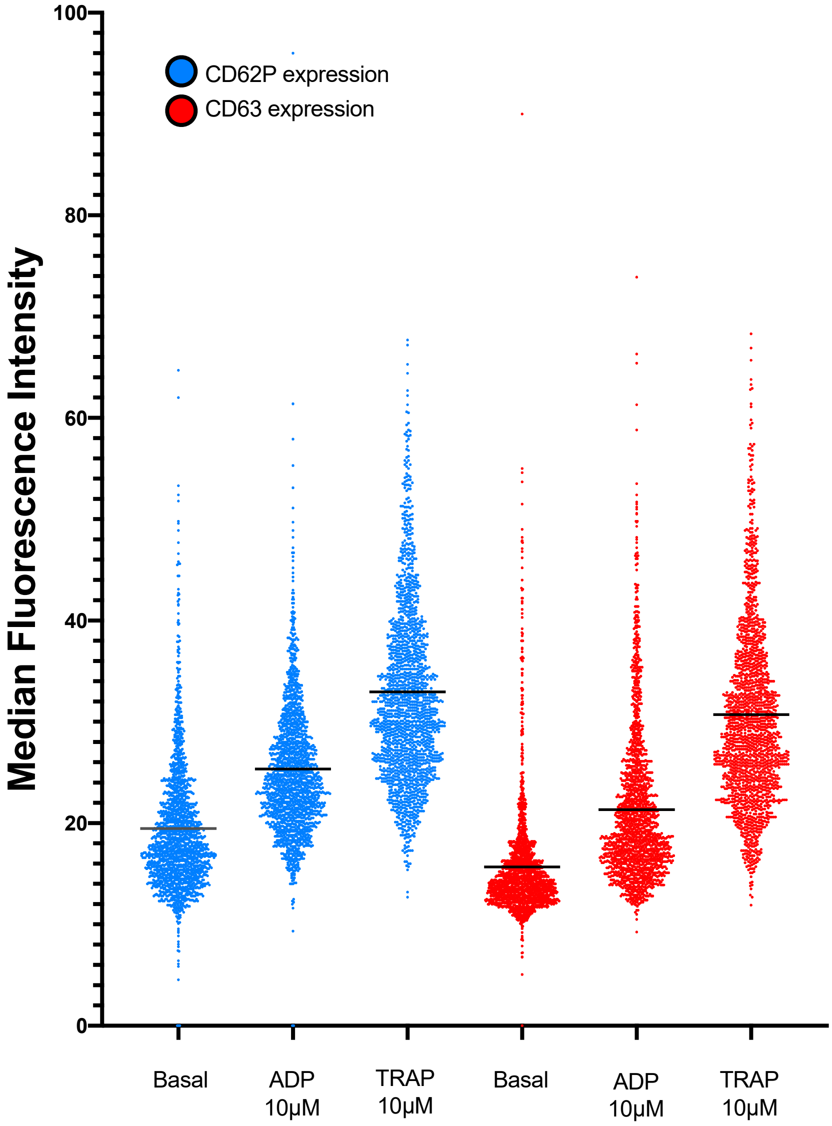

Figure S7: Scatter-plot of platelet CD62P and CD63-expression in response to ADP 10μM and TRAP 10μM stimulation.

Figure S8: Association between glomerular filtration rate, HbA1c and hsCRP with regards to cardiovascular and all-cause mortality in patients with altered platelet reactivity and the reference group

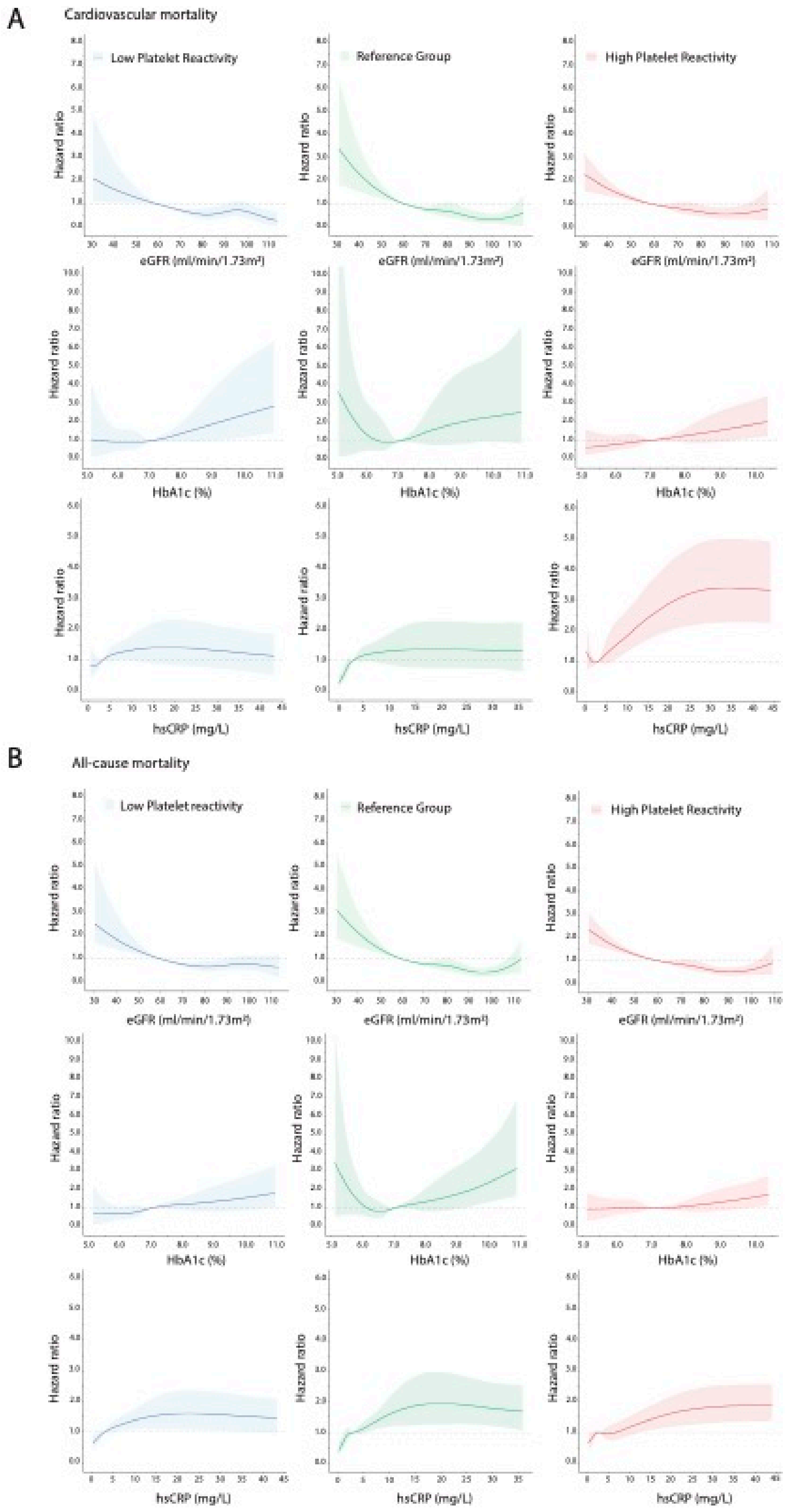

Figure S8: Prediction function of cox models for 464 patients in the Low platelet reactivity group, 545 in the Reference group and 511 in the High platelet reactivity group to assess the continuous relationship between HbA1c, hsCRP and eGFR on cardiovascular (A) and all-cause mortality (B). All continues variables were modeled with restricted cubic splines. Reference values were based on ESC guidelines for cardiovascular disease prevention and observational studies (eGFR: 60ml/min, HbA1c: 7% and hsCRP 3mg/L). The dark lines indicate the hazard function while the shaded area denotes the 95% confidence interval. Each model was adjusted for age and sex (model 1).

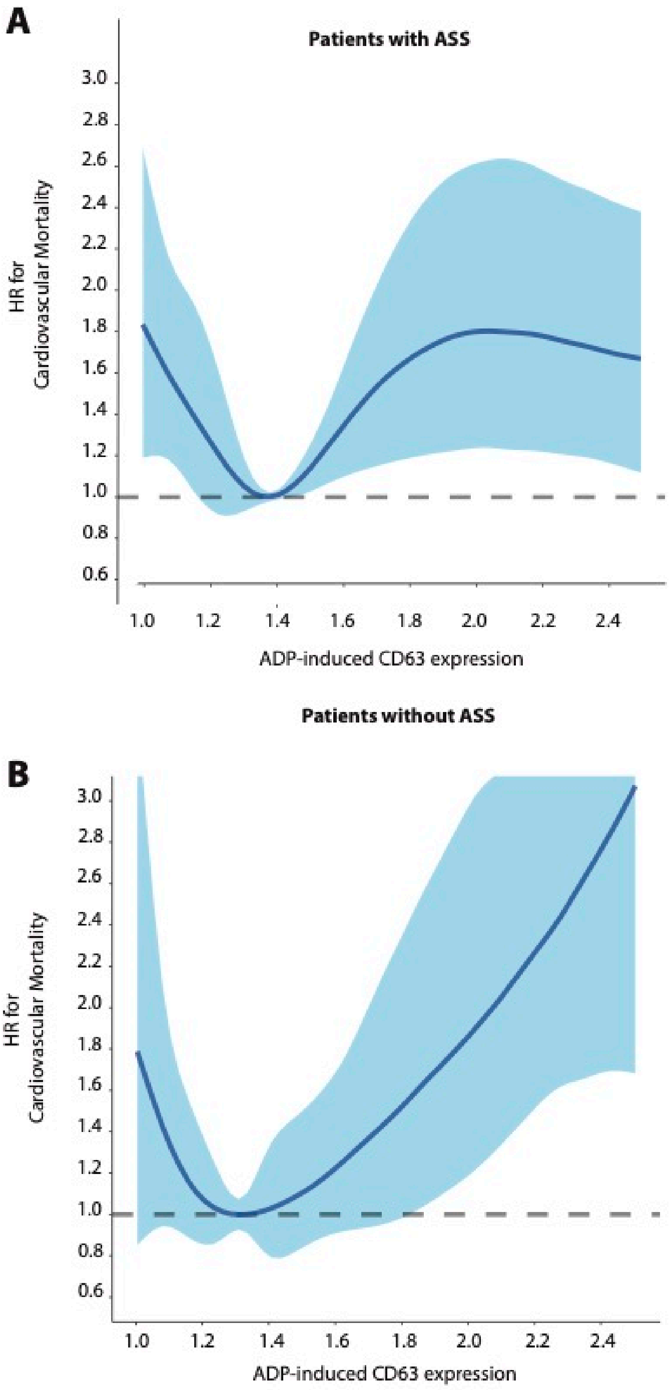

Figure S9: Platelet reactivity and cardiovascular mortality in the LURIC study. Association between ADP-induced CD63 expression and cardiovascular and all-cause mortality in patients with Aspirin therapy (A) and without Aspirin therapy (B).
